# Surgical management of common bile duct stones following repeated endoscopic failure: A retrospective cohort study covering the pandemic period

**DOI:** 10.12669/pjms.41.6.10126

**Published:** 2025-06

**Authors:** Sefa Ozyazici, Ahmet Baris Dirim

**Affiliations:** 1 Sefa Ozyazici Department of General Surgery, Adana City Training and Research Hospital, Turkey; 2 Ahmet Baris Dirim Department of Gastroenterological Surgery, Adana City Training and Research Hospital, Turkey

**Keywords:** Choledochoduodenostomy, Choledocholithiasis, Common Bile Duct Stones, Covid-19, Surgical Outcomes

## Abstract

**Objective:**

Open Common Bile Duct Exploration (OCBDE) is a critical intervention for managing complex common bile duct (CBD) stones when endoscopic retrograde-cholangiopancreatography (ERCP) is unsuccessful. The choice of technique for choledochal exploration remains debated due to variable recurrence rates and complications associated with different methods. This study evaluates the clinical outcomes: bile leakage, oncogenic transformation, and stone recurrence of Choledochoduodenostomy (CDD) versus T-tube drainage (TTD), aiming to substantiate the selection of an optimal CBD closure strategy after choledocholithotomy.

**Methods:**

We conducted a retrospective review of 138 patients who underwent OCBDE between January 1, 2016, and August 1, 2021, at our institution, following failed at least two ERCP interventions. Patients were stratified into two cohorts based on the surgical technique employed: CDD (Group-I) and TTD (Group-II). Clinical and surgical outcomes were meticulously compared between the groups.

**Results:**

Group-I (CDD) included 109 patients (79%), while Group-II (TTD) comprised 29 patients (21%). Bile leaks were identified in 8 patients (5.8%), predominantly in Group-I (p=0.871). In the section of the study encompassing the 1.5-year period of the COVD-19 pandemic, the number of OCBDE surgeries performed remained consistent with the 3.5-year period preceding the pandemic. During the pandemic period, the number of operations increased, while the frequency of TTD surgery decreased (p<0.001). Two patients from Group-I developed bile duct malignancy during follow-up period (p=0.286). Higher incidence of stone recurrence was noted in Group-II (p=0.007).

**Conclusion:**

This investigation delineates the surgical outcomes of side-to-side diamond-shaped CDD compared to TTD, incorporating critical data from approximately 1.5 years of the COVID-19 pandemic. These findings are pivotal for guiding surgical strategy in complicated CBD stone management.

## INTRODUCTION

The prevalence of common bile duct stones (CBDS) in individuals presenting with symptomatic cholelithiasis ranges from 5% to 33%.^1^ In contemporary clinical practice, endoscopic retrograde-cholangiopancreatography (ERCP) is routinely utilized to mitigate complications such as acute pancreatitis, cholangitis, and obstructive jaundice stemming from CBDS.^2^ With the advent of minimally invasive strategies, laparoscopic cholecystectomy post-endoscopic sphincterotomy is recommended for managing CBDS.^3^ Nonetheless, when ERCP fails-owing to issues like large, multiple, impacted stones, previous gastric surgeries, or challenges in cannulating the ampulla-surgical exploration of the common bile duct becomes necessary.^4^

At our institution, a tertiary care center, ERCP is standard for all patients diagnosed clinically and radiologically with CBDS. However, a paucity of literature exists regarding the preferred surgical intervention in cases of recurrent ERCP failure.^5^ In our clinical approach, it is believed that surgical intervention is necessary in cases where CBDS extraction has failed following at least two ERCP attempts. Open Common Bile Duct Exploration (OCBDE) is a procedure that is performed in surgical clinics that do not possess surgical inventory, such as a laparoscopic choledochoscope. The majority of surgical procedures carried out by our institution are open choledochoduodenostomy (CDD) and T-tube drainage (TTD) surgeries.

This study aimed to address the paucity of recent publications on the outcomes of open surgical approaches for complex CBDS management that does not respond to endoscopic interventions, covering the COVID-19 pandemic period.

## METHODS

This retrospective single center cohort study involved 138 patients who underwent open common bile duct exploration between January 1, 2016, and August 1, 2021. Patients who underwent CDD surgery were classified as Group-I, and patients who underwent TTD surgery were classified as Group-II.

### Ethical considerations:

All procedures involving human subjects were performed in accordance with the ethical standards of the institutional and national research committee, the 1964 Helsinki declaration, and its later amendments.This study adheres to the STROCSS criteria for reporting cohort studies in surgery.^7^

### Ethical Approval:

It was granted by the Adana City Research and Training Hospital Institutional Review Board (Approval No: 88/1548, date: 16.09.2021). Informed consent was obtained from all individual participants involved in the study.

These patients were selected based on their benign obstructive jaundice associated with complex CBDS, following unsuccessful ERCP attempts or refusal to undergo ERCP.

### Inclusion & Exclusion Criteria:

The inclusion criteria stipulated that patients must have attained the age of 18 years or more, have undergone open surgery, and have exhibited a common bile duct (CBD) diameter of 0.8 cm or more. Patients whose medical records could not be reached during the postoperative (PO) process, who had a initial diagnosis of malignancy, or who had undergone surgery due to traumatic or iatrogenic injury, were excluded.

### Data collection and analysis:

Comprehensive retrospective data were gathered from electronic hospital record and national health databases, including patient demographics, imaging results (abdominal ultrasonography ± magnetic resonance cholangiopancreatography), ERCP reports, characteristics of CBDS, CBD diameter, American Society of Anesthesiologists (ASA) score, prior surgeries, and the status during the COVID-19 pandemic. The period was dichotomized into pre-pandemic (before March 11, 2020) and pandemic phases. Further data on postoperative (PO) morbidity and mortality, length of hospital stay, average blood loss, operation time, and reasons for readmission were analyzed to compare the outcomes between the two surgical procedures.

The first 30 days period was taken into account for complications due to the operation. The bile leakage classification was evaluated according to the criteria of the international study group of liver surgery (ISGLS).^6^

### Surgical procedure:

**Fig.1:**
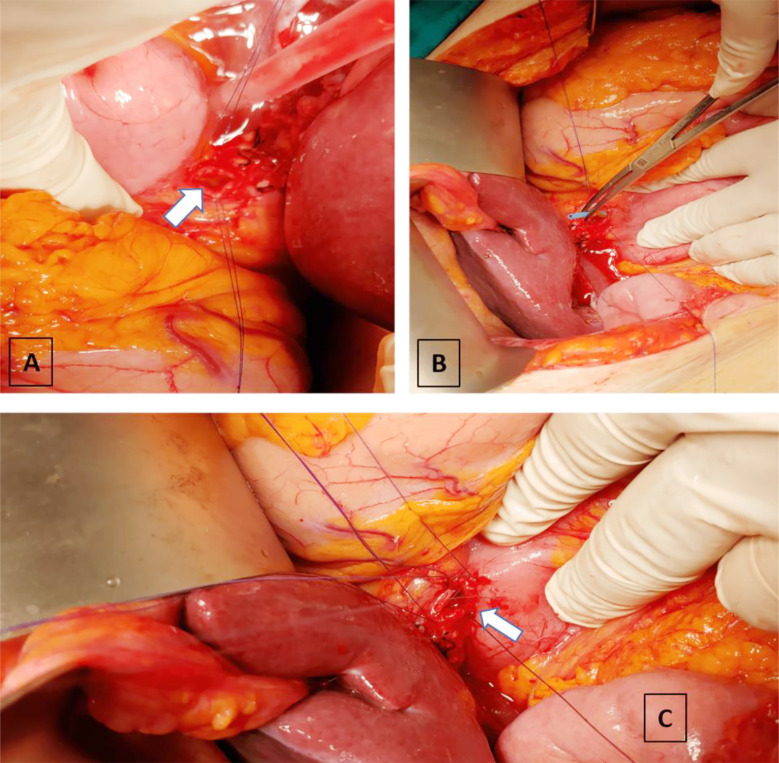
**A)** Two stay sutures placed on the anterior surface of the common bile duct for traction and choledochotomy was performed **B)** Stent extraction **C)** Anterior opening of the side to side choledochoduodenostomy anastomosis.

**Fig.2:**
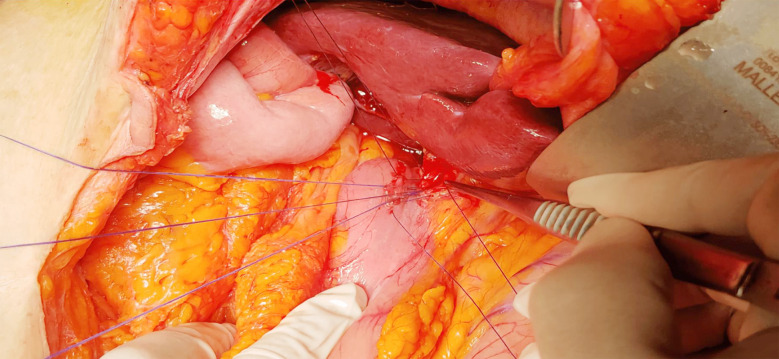
Diamond shaped side-to-side choledochoduodenostomy anastomosis.

OCBDE was performed using a standardized approach. For CDD surgery, a midline incision used to expose the anterior aspect of the CBD, followed by cholecystectomy if not previously performed. A limited Kocher manoeuvre is performed to reduce the tension of the duodenum to be used for duodenal anastomosis. Duodenotomy is performed longitudinally to the 1st continent and biliary and duodenal incisions lie perpendicular to each other for creating diamond shaped anastomosis. Key steps included the placement of two 4/0 silk or vicryl sutures for traction at the lower end of the CBD, a 2 cm longitudinal choledochotomy, debris removal with Randall forceps, and saline irrigation,Fig.1. The bile ducts were examined intraoperatively for retained stones or other lesions using fluoroscopy-guided cholangiogram and/or choledochoscope before creating a diamond-shaped side-to-side anastomosis with interrupted vicryl 4/0 sutures. Fig.2.

For the TTD group, a similar initial approach was followed by the placement of a suitably sized rubber T-tube after stone and debris clearance. The procedure aimed to facilitate future biliary access and manage potential complications. For both surgical procedures frozen pathology was studied in case of suspicion for malignancy. A Jackson-Pratt drain was positioned laterally to the anastomosis, targeting Morrison’s pouch.

### Postoperative management:

Postoperatively, patients with significant comorbidities were monitored in the intensive care unit (ICU). The nasogastric tube was removed on the first postoperative day (POD) once no residual gastric content was confirmed, and the initiation of gas discharge was observed. The drain of Group-I patients who had undergone CDD was removed if there was no bile leakage or hemorrhage, waiting for its content to decrease under 30 cc.

For the Group-II patients t-tube cholangiography was performed on the seventh day; if results were normal, the t-tube was clamped, transitioning the patient to either outpatient care or continued inpatient observation. The drain was removed when bile leakage was ruled out, typically within a few days post-normal cholangiography.

### Statistical analysis:

Data analysis was conducted using SPSS software (Version 23.0, SPSS Inc., Chicago, IL, USA). Continuous variables were tested for normality using the Kolmogorov-Smirnov or Shapiro-Wilk tests; normally distributed variables were described using mean ± standard deviation, while non-normal variables were reported as medians. The Mann-Whitney U test was employed to compare non-normally distributed variables, and categorical outcomes were analyzed using the Chi-square or Fisher’s Exact Test as appropriate. A p-value of less than 0.05 was considered statistically significant.

## RESULTS

In this cohort of 138 patients undergoing surgery for choledocholithiasis, 109 (79%) were assigned to Group-I (CDD) and 29 (21%) to Group-II (TTD). The primary indications for surgery included large stones in 18 patients (13%), multiple stones in 41 (29.7%), impacted stones in 43 (31.2%), ERCP failures in 26 (18.8%), and cholangitis in 10 (7.2%). When the indications were detailed, there was a statistically significant difference between the two groups in terms of multiple stones and impacted stones (p=0.001, p=0.001 respectively), detailed further in Table-I. ERCP failures were primarily due to anatomical challenges such as bulbary diverticulum or apical stenosis, and physiological alterations post-bariatric bypass surgery or antrectomy for ulcers, which have been increasingly noted in recent years. Detailed reasons for ERCP failures are cataloged in Table-II.

**Table-I:** Comparative demographic and clinical characteristics of patients who underwent open common bile duct exploration.

	Total n:138	Group-I (CDD) n: 109 (79%)	Group-II (TTD) n: 29 (21%)	P-value
Age, mean year (±std)	61.6±15.1	62.9±14.9	56.8±15.4	0.047
***Gender***				0.621
Female	77 (55.8%)	62 (80.5%)	15 (19.5%)	
Male	61 (44.2%)	47 (77%)	14 (23%)	
***Covid-19 Period***				
Normal	67(48.6%)	43 (64.2%)	24 (35.8%)	<0.001
Pandemic	71(51.4%)	66 (93%)	5 (7%)	
***Endications***				0.860
Big stone	18 (13%)	15 (83.3%)	3 (16.7%)	0.001
Multiple Stones	41 (29.7%)	23 (56.1%)	18 (43.9%)	0.001
Impacted stone	43 (31.2%)	42 (97.7%)	1 (2.3%)	1.000
ERCP failure	26 (18.8%)	20 (76.9%)	6 (23.1%)	0.627
Cholangitis	10 (7.2%)	9 (90%)	1 (10.0%)	
***Postoperative Complications***				0.435
Pulmonary	6 (4.3%)	6 (100%)	0 (0%)	0.671
Cardiac	4 (2.9%)	4 (100%)	0 (0%)	0.318
Wound site infection	14 (10.1%)	13 (92.9%)	1 (7.1%)	0.871
Bile leakage	8 (5.8%)	7 (87.5%)	1 (12.5%)	0.671
Mortality	4 (2.9%)	3 (75%)	1 (25%)	0.475
Pancreatitis	1 (0.7%)	1 (100%)	0 (0%)	0.475
Cholangitis	1 (0.7%)	1 (100%)	0 (0%)	0.851
Sepsis	3 (2.2%)	2 (66.7%)	1 (33.3%)	0.851
Hematoma	3 (2.2%)	3 (100%)	0 (0%)	
***ASA Score^a^***				0.435
I	6 (4.3%)	6 (100%)	0 (0%)	0.716
II	54 (39.1%)	44 (81.5%)	10 (18.5%)	0.675
III	69 (50.0%)	53 (76.8%)	16 (23.2%)	0.606
IV	9 (6.5%)	6 (66.7%)	3 (33.3%)	
***Preoperative Surgeries***				0.964
Cholecystectomy	40 (29%)	31 (77.5%)	9 (22.5%)	0.806
Gastric surgery	6 (4.3%)	4 (66.7%)	2 (33.3%)	0.475
Laparotomy	1 (0.7%)	1 (100%)	0 (0%)	0.475
T-tube decompression	1 (0.7%)	1 (100%)	0 (0%)	
CBD diameter, median mm (min-max)	12 (8-30)	12 (8-30)	13 (9-22)	0.049
Follow-up period, median month (min-max)	18 (0-84)	13(0-79)	45 (10-84)	<0.001
Operation time, median minute (min-max)	105 (80-180)	100 (80-180)	113 (100-160)	0.001
Blood loss, median ml (min-max)	80 (40-200)	90 (40-200)	80 (50-100)	0.003
Length of hospital stay, median day (min-max)	8 (2-42)	8 (2-42)	11 (7-33)	<0.001

ASA, American society of anesthesiologists;

a Score I: normal healthy person, II: mild systemic disease, III: severe systemic disease, IV: severe systemic disease that is a constant threat to life.

CDD, choledochoduodenostomy, CBD, common bile duct

**Table-II:** Reasons for ERCP failure.

	Total	Group-I (CDD)	Group-II (TTD)	P-value
Gastric surgery	6 (4.3 %)	4 (66.7 %)	2 (33.3 %)	0.899
Bulbary apical stenosis	9 (6.5 %)	6 (66.7 %)	3 (33.3 %)	0.679
Citus in versus	1 (0.7%)	1 (100 %)	0 (0 %)	0.514
Ampullary diverticulum	5 (3.6 %)	4 (80 %)	1 (20 %)	0.682

Fisher’s exact test applied.

**Table-III:** Reasons for readmission in the follow up period.

	Total	Group-I (CDD)	Group-II (TTD)	P-value
Jaundice	11 (8 %)	1 (9.1 %)	10 (90.9 %)	0.007
Malignancy	2 (1.4 %)	2 (100 %)	0 (0 %)	0.286
Cholangitis	2 (1.4 %)	1 (50 %)	1 (50 %)	0.648
Pancreatitis	2 (1.4 %)	2 (100 %)	0 (0 %)	0.286
Abscess	2 (1.4 %)	1 (50 %)	1 (50 %)	0.648
Persistant bile leakage	1 (0.7 %)	1 (100 %)	0 (0 %)	0.833

Fisher’s exact test applied.

Bile leakage occurred in eight patients (5.8%), more frequently in Group-I. Six of these cases were managed conservatively (one patient’s drain, which was inadvertently breached the CDD anastomosis line on gastroscopic control, was withdrawn). The other 51 years old patient’s CDD anastomosis was revised with a successful Roux-en-Y hepaticojejunostomy (HJ) on the 17th postoperative day due to a Grade-C fistula. Moreover, this patient was later diagnosed with unresectable peritoneal carcinomatosis, indicating advanced malignancy during a follow-up laparotomy eight months post-operation. Another CDD patient experienced anastomotic insufficiency and underwent successful primary repair on postoperative day 10, with no subsequent complications reported.

The study period included approximately 1.5 years of the COVID-19 pandemic, during which 71 patients (51.4%) underwent surgery, compared to 67 patients (48.6%) in the four years prior. The increase in surgical interventions during the pandemic period was statistically significant (p<0.001). Within the first month post-operation, four patients (2.9%) succumbed to complications; two due to cholangiosepsis, one following cardiac complications on postoperative day two, and another due to complications from a subhepatic abscess and wound evisceration 14 days after surgery.

Two patients (1.4%) in Group-I developed malignancy during the follow-up. The first was re-operated for a biliary fistula and later diagnosed with biliary malignancy. The second presented with worsening jaundice ten months post-discharge; however, definitive surgery was precluded because of the clinical condition.

Among 11 patients presenting with non-malignant jaundice, recurrent stones were identified in 10 Group-II patients during ERCP, necessitating further intervention (p=0.007). HJ revision surgery was performed because ERCP stone extraction failed in one of the Group-II patients. Patients undergoing T-tube drainage experienced longer surgeries, averaging 113 (100-160) minutes (p=0.001). Also the CBD diameter was wider as 13 mm (9-22), the length of hospital stay was longer as 11 days (7-33) and the blood loss was lower as 80 ml (50-100) in the same group (p =0.049, p<0.001, p=0.003 respectively). These details, along with additional demographic and clinical data, are summarized in Table-I.

## DISCUSSION

When ERCP fails to alleviate benign obstructive jaundice, surgical intervention becomes essential.^8^ Post-choledochotomy, the process of stone removal may result in a range of adverse outcomes, including papillary spasm and oedema. This obstruction can impede the normal drainage of bile, leading to a condition known as biliary hypertension. In severe cases, this obstruction can result in bile leakage through the choledochorraphy. The efficacy of the common bile duct drainage method in reducing bile pressure remains a subject of debate. Recent literature has revealed a significant debate with no strong consensus on the optimal method of duct closure to ensure the best possible outcome in terms of biliary complications.^9^

While CDD is linked to potential complications like alkaline reflux gastritis, cholangitis, and sump syndrome, it is preferable due to its minor anatomical disruptions and reduced need for extensive dissection. Sump syndrome, characterized by recurrent cholangitis and subsequent hepatic abscess from debris impaction in the CBD, has been reported with a prevalence of up to 5.2% in similar surgical contexts.^10^ Our adoption of the diamond-shaped anastomosis using interrupted sutures likely contributed to the absence of sump syndrome in our study, as this technique minimizes bile stasis and intra-ductal pressure, facilitating the efficient clearance of bile and debris.^11^

The utilization of T-tubes aims to prevent bile stasis, reduce intrabiliary pressure, and provide a route for percutaneous stone extraction.^12^ In our study, a total of 11 patients (8%) had recurrent calculi consistent with the literature data gives the rate after OCBDE ranging from 1% to 8%.^13^ On the other hand, Sahara et al. reported a retained/recurrent stone rate of 18% in patients who had undergone choledocholithotomy and t-tube drainage for primary choledochal stones.^10^ It is statistically significant that 10 of these patients were in TTD Group-2. In our study, we present the average follow-up time of patients, which is not available in the current literature data.^9^ The mean duration of follow-up in the TTD group was 45 months (10-84), which is statistically significantly longer than the CDD group. The incidence of recurrent calculi may have been higher in the TTD group due to the longer follow-up period.

Biliary stricture constitutes a significant concern for patients undergoing OCBDE. In accordance with the findings of the majority of studies conducted on this subject, the CBD stricture rate was found to be non-existent. The primary risk factor associated with biliary stricture was found to be a small CBD diameter.^14^ In order to circumvent this issue, it is recommended that an optimal CBD diameter of a minimum of 8-10 mm be employed for a safe choledochotomy, as evidenced by the findings of our study.

Post-biliary enteric anastomoses often lead to duodenal content regurgitation into the biliary tract, escalating the risk of inflammatory processes and potentially increasing the likelihood of bile duct carcinoma.^15^ As an advantage of open surgery, distal CBD and pancreatic head can be palpated with generous duodenal coherization and frozen pathology including tru-cut biopsy can be studied in doubtful cases. H Okamato et al. reported a 1.9% incidence of malignancy after choledochojejunostomy and 7.6% after CDD.^16^ Our result was inconsistent with this finding, we think that our low incidence results from mandatory intraoperative confirmation by cholangiogram and choledochoscope. While no malignancy was detected in Group-II patients in our study, it was detected in 2 (1.4%) patients during the follow-up period of Group-I patients.

The COVID-19 pandemic significantly impacted surgical practices, with a sharp increase in the number of operations performed during this period compared to the years prior. This surge likely reflects the escalated severity of cases, as suggested by Nadell et al, who noted an increase in severe acute cholecystitis during the pandemic.^17^ The increase in the number of patients who had more severe acute cholecystitis during the pandemic period may have caused an increase in the CBDS rate, as we reported. There was no Covid-19 pcr test positivity in any of the small number of pulmonary complications that developed in both groups of patients who were operated during the pandemic period.

Our study’s bile leakage rates, were noteworthy but some extent is consistent with existing literature, leakage was detected in eight patients (5.8%), although it was not statistically significant, it was higher in Group-I (seven patients). Bile leakage in six patients was regressed with conservative follow-up, while Grade-C fistulas in two patients required revision surgeries. In patients undergoing CDD surgery, Jalal et al.^18^, Leppard et al.^19^ and Asad et al.^20^ reported a bile leakage rate of 18%, 13%, and 2.3%, respectively. Post-removal of the T-tube, biliary leakage has been documented as the most prevalent complication, with incidence rates ranging from 3.8% to 6.9%.^21^

### Strength and Limitation:

It is hypothesised that the fact that all surgical interventions were performed by the same experienced surgical team, who have completed the learning curve, is significant in terms of homogeneity of the results. Furthermore, the fact that it has a longer follow-up period than the studies presented in the literature, that it includes open common bile duct exploration data related to the older population, and that it covers the period of the Covid pandemic are all significant. The present study is subject to several limitations. Firstly, it should be noted that this is a retrospective single-centre study. Secondly, laparoscopic surgery is not available at this centre.

## CONCLUSION

The side-to-side diamond-shaped choledochoduodenostomy demonstrated a lower incidence of cholangitis and recurrent stones compared to TTD surgery, suggesting its efficacy in complex CBDS management. However, patients undergoing bilioenteric drainage surgeries require close monitoring in the postoperative period for potential complications, including biliary tract malignancy.

### Authors’ contributions:

**SO:** Design of the study, critical revision of the paper.

**ABD:** Acquisition of data, analysis & interpretation of data.

All authors have read and approved the final version of the manuscript.
